# Effectiveness of a telemonitoring intensive strategy in early rheumatoid arthritis: comparison with the conventional management approach

**DOI:** 10.1186/s12891-016-1002-2

**Published:** 2016-04-02

**Authors:** Fausto Salaffi, Marina Carotti, Alessandro Ciapetti, Marco Di Carlo, Stefania Gasparini, Sonia Farah, Marwin Gutierrez

**Affiliations:** Rheumatology Department, Polytechnic University of the Marche, Jesi, Ancona, Italy; Radiology Department, Polytechnic University of the Marche, Ancona, Italy; Rheumatology Department, Betsi Cadwaladr University Health Board, Glan Clwyd Hospital, Bodelwyddan, Denbighshire, Wales; DII, Department of Information Engineering, Politechnic University of Marche, Ancona, Italy; Musculoskeletal Department, National Rehabilitation Institute, Mexico City, Mexico; Clinica Reumatologica, Università Politecnica delle Marche, c/o Ospedale “Carlo Urbani”, Via Aldo Moro, 25, 60035 Jesi, AN Italy

**Keywords:** Telemonitoring, Rheumatoid arthritis, Disease activity, Healthcare

## Abstract

**Background:**

The advent of Internet and World Wide Web has created new perspectives toward interaction between patients and healthcare professionals. Telemonitoring patients with rheumatoid arthritis (RA) is an emerging concept to guide the collaborative management treatment and improve outcomes in patients. The objective of this study was to investigate whether an intensive treatment strategy, according to a telemonitoring protocol, is more effective than conventional management strategy in reaching remission and comprehensive disease control (CDC) after 1 year in early rheumatoid arthritis (ERA) patients.

**Methods:**

Forty-four ERA patients were randomly allocated into two groups: the telemonitoring intensive strategy (TIS) group (group 1) or the conventional strategy (CS) group (group 2). Three patients refused to participate. In group 1 (*n* = 21), a remote monitoring system of disease activity, in combination with protocolised treatment adjustments aiming for remission was applied. In group 2 (*n* = 20), patients were treated according to daily clinical practice, with regular evaluation of disease activity, but without protocolised treatment adjustments. A telemedical care called “REmote TElemonitoring for MAnaging Rheumatologic Condition and HEaltcare programmes” (RETE-MARCHE), was developed to perform the remote monitoring.

**Results:**

A higher percentage of patients in the TIS group achieved CDAI remission vs patients in the CS group (38.1 % vs 25 % at year 1, *p* <0.01). Time to achieve remission was significantly shorter in the group 1 than in the group 2, with a median of 20 weeks vs a median over 36-weeks (*p* <0.001). Concordantly, the patients in group 1 showed a greater improvement (*p* <0.001), compared with group 2 in terms of functional impairment (71.4 % vs 35 %) and radiological damage progression (23.8 % vs 10 %), resulting in a greater rate of CDC (19.4 % vs 5 %).

**Conclusions:**

According to our results, an intensive treatment strategy by telemonitoring leads to more effective disease remission and more rapid CDC than treatment according to conventional management strategy in ERA.

**Trial registration:**

Trial registration number: ISRCTN13142685

Date of registration: March, 17^th^ 2016

## Background

Rheumatoid arthritis (RA) is the most frequent chronic inflammatory joint disease, affecting 0.4–1 % of the population [1]. It is characterised by a progressive inflammation leading to irreversible joint damage that reduces the function, increases the disability and may determine a poor prognosis, influencing health care resources and budgets. In the last years, the introduction of targeted biologic therapies has greatly improved the management of RA [2, 3]. A tight control of the disease, including a “treat-to-target” (T2T) approach, has demonstrated positive advantages [4]. This strategy, aimed for low disease activity (LDA) or remission seems even more important than specific therapies adopted [4–6]. Moreover, a tight control assessment of the disease may stabilize patients reducing the need for hospitalization. Although the T2T concept is currently highly accepted by the RA community [7] it still represents a challenge in daily clinical practice due to some aspects such as: (a) a proportion of RA patients is treated by rheumatologists who have no confidence with the T2T, (b) not all rheumatologists adopt the T2T strategy in their routine care, (c) the correct T2T approach requires repetitive patient clinical assessments and regular application of disease activity measurements that are not always available in the busy daily practice [7].

Despite the availability of a high number of patient-reported outcome (PRO) measures and its documented utility to assess accurately the disease progression and/or the responsiveness and to evaluate the physical and the psychosocial problems [8, 9], their application in the routine practice is limited since they require a manual data computation which is time consuming and can be a source of error. To overcome these problems, researchers are working on alternative tools to traditional paper-based instruments. The available interactive electronic systems have opened a variety of opportunities that permit a closer involvement of the patient in planning, implementation and evaluation of their care. The most remarkable systems use an office touch-screen computer, internet-based approach [10, 11], telephone-based interactive voice-response system [12], handheld computer [13] or mobile phone [12]. Web-based home telemonitoring is an appealing strategy of telemedicine in which physiological and clinical data are transferred from the patients’ home to the telemonitoring center facilitating the tight control of the patients, the interpretation of the clinical data and the therapeutical decisions [14–18].

In many health care systems around the world, Web based home telemonitoring is already an integral part of a broader view and represents the most promising application of telemedicine for delivering cost effective quality care [18, 19]. Taking into account the promising field of telemonitoring, we decided to test this system in a tailor-made disease specific and self-care support. To date, little is reported on the effects of knowledge and self-care using the telemonitoring systems [20, 21]. So, we addressed this study to investigate whether an intensive treatment strategy, according to a specific protocol by a telemonitoring solution is more effective than a treatment according to conventional management approach in reaching remission and comprehensive disease control (CDC) [22] after 1 year of follow-up in early RA (ERA) patients. Additionally, we determined the degree of patients’acceptance of the telemonitoring platform.

## Methods

### Design and study population

Forty-four ERA patients from Rheumatology Clinic of the Università Politecnica delle Marche (Italy), were randomly allocated to one of two strategy groups: the telemonitoring intensive strategy (TIS) group (group 1) or the conventional strategy (CS) group (group 2). In the group 1, an intensive remote control approach of disease activity, in combination with protocolised treatment adjustments aiming for remission was applied. In the group 2, patients were treated according to a more liberal therapeutic protocol: although strict in terms of assessment timing, dose adjustments were based on the opinion of the single rheumatologist. ERA patients were randomised 1:1 by a computer generated randomization list prepared by biomedical engineer (SF) uninvolved in the clinical conduction of the trial, who allocated the assigned group when called by the clinical investigators. Clinical investigators were blinded to the allocation sequence. After group allocation, it was no more possible to blind the clinical investigators or the study partecipants to the strategy actually receveid. Three patients (one of the group 1 and two of the group 2) did not accept to participate to the study due to difficulties in reaching the centre, so they were excluded. All RA patients were included according to the following inclusion criteria: diagnosis according the 2010 ACR/EULAR criteria for RA [23], age ≥18 years, disease duration less than 1 year (the disease duration was considered from the onset of the symptoms to baseline that corresponded with the point of diagnosis and with the start of treatment), and Clinical Disease Activity Index (CDAI) ≥22 [24–27]. Patients were excluded if visual limitations were present, they were hard of hearing in combination with living as a single person, did not have command of the Italian language, and/or suffered from diseases requiring hospitalization such as chronic obstructive pulmonary disease, heart disease, multiple sclerosis, extracorporeal dialysis or chronic infectious disease. Patients needing biologic therapy were screened for tuberculosis prior to treatment and those at high risk for tuberculosis were allowed to enter in the study after chemoprophylaxis, as local recommendations [28]. All patients agreed to be enrolled and provided written informed consent. The Hospital Clinic ethics committee (Comitato Unico Regionale – ASUR Marche) approved the study.

### The TIS group (group 1)

All 21 patients enrolled in this group performed a total of 13 visits: 5 face-to-face visits (at baseline, 3, 6, 9 and 12 months of follow-up) and 8 televisits (at 1, 2, 4, 5, 7, 8, 10 and 11 months). After the diagnosis, patients started a therapeutic preset schedule beginning with methotrexate (MTX) 15 mg/week. Folic acid was prescribed to every patient taking MTX (0.5 mg/day). In case of no improvement, the consecutive intensification steps with a disease modifying anti-rheumatic drug (DMARD) were adopted: at month 2, if needed an additional visit was performed and the dose of MTX was increased to 20 mg/week; at month 3 in case of still scarse response, sulphasalazine (SSZ) 2000 mg/day orally was added. A further step if the target has not been reached at month 6 was the introduction of a biologic treatment administered subcutaneously as follow: adalimumab (ADA) 40 mg on alternative weeks, etanercept (ETN) 50 mg once a week, abatacept (ABA) 125 mg once a week [29, 30]. Each one was associated with MTX if no contraindications were present. Non-steroidal anti-inflammatory drugs (NSAIDs), prednisolone at ≤10 mg/day and intra-articular corticosteroid injections were allowed at the discretion of the attending rheumatologist.

### The CS group (group 2)

A total of 20 patients were enrolled in this group. All patients were regularly assessed at baseline, 3, 6, 9 and 12 months. Here, the treatment decisions were made at any visit according to the discretion of the rheumatologist. In this group, patients have been visited every three months in the outpatient clinic and treated with conventional DMARDs and/or biological drugs following the guidelines for RA [28–30]. At every visit, the theraphy was suited to the single patient by the rheumatologist evaluator. A common strategy was to start with MTX (15 mg/week); in case of inefficacy, other DMARD or biologic agents could be added. This group represent a “real life” sample of the common clinical practice in our third-level centre of referral in ERA patients.

The main differences between the groups were two: first, in group 1 has been used an accurate route of step-up therapy, second, the traditional face-to-face visits have been associated with the innovative televisits, that have allowed a more frequent, although remote, monitoring of the disease activity. A similar kind of approach, with the distinction in a conventional strategy group and an intensive strategy group, has been already described in the CAMERA study [31].

### Telemonitoring system

**Fig. 1:**
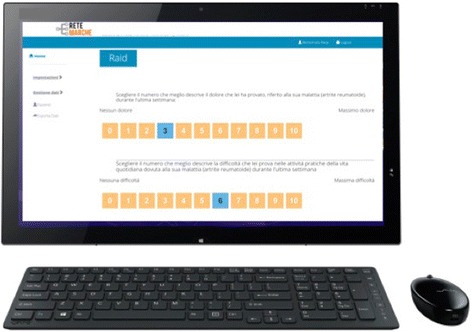
The graphical interface of the RETE-MARCHE provides a quick overview of the navigation process. The questions were answered by selecting one of the radio buttons on the screen

A telemedical care called “REmote TElemonitoring for MAnaging Rheumatologic Condition and HEaltcare programmes” (RETE-MARCHE), was developed to perform a remote monitoring. It is a specialized Website platform constructed to reduce the risk of keying errors and to be cost-effective. The Web portal (http://www.rete-marche.it/healthcare/) allows at the authorized users to access into the system by a personal computer. The graphical interface provides a quick overview and supports straightforward navigation. After the registration, the system generates some questions which must be answered by selecting one of the radio buttons proposed on the screen (Fig. 1). To ensure privacy and accessibility, each user receives a username and password to access via the Web/Internet portal. Each question must be completed before the system continues to the next screen. For obvious reasons, in our study, the questions and animations of the electronic system were presented in the Italian language. Alternatively, the system is programmed for multiple languages. Prior to proceeding with compilation of the computerized questionnaires, all patients received a brief training session to familiarize with the personal computer components and the technical aspects. In addition, a real-time trained facilitator was available within the system to provide procedural assistance in case of need. Furthermore, a help desk accessible via phone or email was established during the study. The patients variables recorded in the Web site platform included the following information: demographic data and the patients’ 11-number button numeric rating scale (NRS) format for RA Impact of Disease (RAID) (Fig. 1). The RAID score is developed, translated and validated across several countries; it is free of charge and fast, making it feasible and widely applicable [32, 33]. It measures seven domains, each with 0–10 NRS that are perceived by patients to be particularly important for their health. Each domain has the following weight: pain 0.21, functional disability 0.16, fatigue 0.15, sleep problems 0.12, emotional well-being 0.12, physical well-being 0.12 and coping 0.12. The score has a range from 0 to 10 (10 worst health). This score can be used in clinical trials as a new composite index that captures information relevant to RA patients. Like a push technology, the computer system generated warnings to both the patient and the clinician case manager whenever it detected that the patient’s self-monitoring showed deterioration in one or more of the symptoms monitored with RAID. Specifically, if the RAID did not show an improvement in the first two months of treatment at least 30 % (relative improvement) or 2 points (absolute improvement) and then, from the third month onwards at least a 50 % (relative improvement) or 3 points (absolute improvement) from baseline (see RAID validation study) in such situations, the system immediately intervened, sending pre-programmed advice on the appropriate required action [34]. At the same time, the clinical case manager was automatically notified of the problems and the suggestions made to the patient. The case manager could therefore monitor the patient’s responses at distance and, if necessary, directly intervene by phoning the patient or informing the attending physician, who determined an appropriate response and patients were encouraged to return for a follow-up visit at the Clinic for any treatment modification. At the end of the study, electronically collected raw data – including number, age and gender of participating patients as well as duration of assessments and test results produced by system were extracted from practice computers and pseudonymised. This information was available to the telemonitoring clinical team.

### Assessment of variables

Each clinical evaluation included an assessment of disease activity and function by using CDAI [24] and Recent-Onset Arthritis Disability index (ROAD) questionnaire, respectively [35, 36]. Erythrocyte sedimentation rate (ESR – millimetre per first hour), C-reactive protein (CRP – milligram per litre), rheumatoid factor (RF) and anti-citrullinated protein antibodies (ACPAs) status were defined at baseline. The CDAI omits the CRP and is based on the simple summation of the count of swollen/tender joint count of 28 joints along with patient and physician global assessment on visual analogue scale (VAS 0–10 centimeters) scale for estimating disease activity [24]. The values can range from 0 to 76 [26]. High disease activity is defined as a CDAI greater than 22, moderate activity as a CDAI greater than 10 and less or equal to 22, low activity as a CDAI less or equal to 10 and greater than 2.8, and remission as a CDAI less or equal to 2.8 [26, 27]. CDAI will prove to be of the greatest value in clinical practice rather than research, where acute-phase reactants are nearly always available. The greater advantage associated with CDAI is its potential to be employed in evaluation of patients with RA consistently with close frequency and independently of any calculating device, therefore, it can essentially be used everywhere and anytime for disease activity assessment in RA patients [25–27]. The ROAD comprises 12 items that capture a combination of common symptoms related to a patient’s level of functional ability and includes important questions concerning upper extremity function, lower extremity function, and activities of daily living/work. For each item, patients are asked to rate level of difficulty over the past week on a 5-point scale, which ranges from 0 (without any difficulty) to 4 (unable to do) [34, 35]. The ROAD ranges from 0 to 48. In order to express these scores in a more clinically meaningful format, a simple mathematical normalization procedure was then performed so that all the scores could be expressed in the range 0–10, with 0 representing better status and 10 representing poorer status [36, 37]. The ROAD can be scored in 15 to 20 seconds. Radiographs of hands and feet were obtained in anteroposterior view and digitized at baseline and after 1 year to follow the disease evolution. X-rays were evaluated by two experienced readers, according to Sharp’s method as modified by Sharp–van der Heijde Score (total Sharp score – TSS) [38]. Both readers were blinded to patient identity, characteristics and treatment. Moreover the X-rays were scored in paired order (without information on the chronology of the films) [39]. For each set of radiographs, the mean score of the two readers was used for the analyses. Thirty-two joints in the hands and 12 in the feet were scored for erosions, with a maximum score of 5 per joint in the hands and 10 per joint in the feet. Joint space narrowing was graded from 0 to 4 in 30 joints in the hands and in 12 joints in the feet [38]. The principal score used in the analyses is the total score, which is the sum of the erosion score and the joint space narrowing score (range from 0 to 448) [38]. The change in the TSS was calculated by subtracting baseline score values from the respective final scores. Absence of radiographic progression was defined as a ≤0.5 unit change from baseline in delta-TSS [22]. A subset of 25 chosen pairs radiographs was read twice, with an interval of at least 2 weeks in order to ascertain precision of the readings (the intraclass correlation coefficient between the two investigators was 0.93).

### Clinical efficacy and radiographic progression

Primary outcome measures consisted of the proportion of patients in clinical remission at 1 year (according the EULAR definition, CDAI <2.8). Besides EULAR remission, characterized by a stringent control of the signs and symptoms of inflammation, we also assessed the simultaneous achievement of normal physical function and the absence of radiographic progression. Normal physical function was assessed using the ROAD and defined as ROAD <1 [36]. Radiographic progression was assessed using radiographs of the hands/wrists and feet scored using the delta-TSS method. Absence of radiographic progression was defined as a ≤0.5 unit change from baseline in TSS. Patients who achieved all three components were considered to have achieved CDC (‘CDC achievers’), while patients who achieved either none or any one or two (partial achievers) of the three components were considered to have not achieved CDC (‘CDC non-achievers’) [22, 30]. Secondary endpoints was the 1-year area under the curve (AUC) of minor, moderate and major CDC response cutpoints (50 %, 70 % and 85 % improvement) [27].

### Patient’s acceptance of telemonitoring system

The patient’s acceptance of telemonitoring system was established by asking the following questions: (a) is the automated telemonitoring system easy to use?; (b) is the automated telemonitoring system easy to understand?; (c) does the information in the telemonitoring system meet your personal needs?; (d) do you find the information in the telemonitoring system useful for managing your own care?; (e) in general, are you satisfied with use of the automated telemonitoring system? For each question, the patient could express their opinion through a scale ranging from 1 to 5 (1 = not at all; 2 = somewhat; 3 = moderately; 4 = very; 5 = enormously). Furthermore, we asked all patients the following question: ''If you could keep the automated telemonitoring system in your home, would you continue to use it in the future?''.

### Statistical analysis

Descriptive statistics are given as mean (SD) and median (95 % confidence interval – CI) for continuous data or as percentages for counts. To compare the baseline differences between groups chi-square test corrected for continuity and Student’s t test were employed. The cumulative inflammatory burden was estimated by the CDAI, expressed in time-integrated values (area under the curve – AUC), calculated for each patient during the 1-year follow-up. Mann–Whitney U test was used to compare AUC by CDAI and difference last-first. The reliability of the radiographic scores was assessed using the intraclass correlation coefficient (ICC). The ICC measures the repeatability of the scores from each reader and the repeatability of the averages of the 2 readers' scores. Wilcoxon’s signed rank test was used to assess differences in TSS between baseline and 1-year follow-up. The Spearman’s correlation coefficient was employed to test the correlation between the scores of CDAI and RAID. A chi-square test corrected for continuity was used to evaluate differences between treatment groups in remission, CDC and CDAI response cutpoints. Statistical analysis was performed using the Statistical Package for Social Sciences (SPSS Inc., Windows release 11.0; Chicago, Illinois, USA), and MedCalc 10.5 (Mariakerke, Belgium) statistical software.

## Results

### Study cohort

The study cohort consisted of 21 patients followed with TIS, with a mean duration of symptoms of 5.9 months and 20 patients followed in CS with a mean duration of symptoms of 6.3 months. All patients had active disease with a mean ± s.d. CDAI of 25.7 ± 6.8, 75.7 % were RF positive and 56.2 % were ACPAs positive. Ten patients in the two groups were on steroids. No statistically significant differences in the demographic and baseline disease characteristics between the two groups were found (Table 1).

**Table 1:** Baseline demographic and disease characteristics

	TIS group *n* = 21	CS group *n* = 20	*P*
Age, mean ± s.d., years	49.3 ± 15.2	50.3 ± 16.3	n.s.
Women, n (%)	16 (76.2)	15 (75.0)	n.s.
Symptom duration, mean ± s.d., months	5.9 ± 2.5	6.3 ± 4.1	n.s.
IgM RF positive, n (%)	15 (71.4)	16 (80.0)	n.s.
ACPAs positive, n (%)	11 (52.4)	12 (60.0)	n.s.
Patient assessment of disease activity (NRS 0–10), mean ± s.d.	6.4 ± 1.3	6.0 ± 1,7	n.s.
Physician assessment of disease activity (NRS 0–10), mean ± s.d.	6.1 ± 1.6	5.7 ± 2.1	n.s.
Tender joint count (0–28), mean ± s.d.	9.0 ± 4.4	7.9 ± 3.1	n.s.
Swollen joint count (0–28), mean ± s.d.	5.5 ± 3.3	4.9 ± 2.5	n.s.
ESR, mean ± s.d.	36.1 ± 22.8	35.9 ± 20.	n.s.
CRP, mean ± s.d., mg/l	32.0 ± 33.4	28.7 ± 26.0	n.s.
CDAI score, 0–76 scale, mean ± s.d.	27.2 ± 3.6	24.3 ± 8.0	n.s.
RAID, score, 0–10 scale, mean ± s.d.	7.5 ± 0.9	6.4 ± 2.5	n.s.
ROAD score, 0–10 scale, mean ± s.d.	4.9 ± 2.14	4.5 ± 2.4	n.s.
Total Sharp Score, 0–448 scale, mean ± s.d.	9.4 ± 4.79	10.8 ± 5.6	n.s.

Abbreviations: *RF* Rheumatoid Factor, *ACPAs* Anti-Citrullinated Protein Antibodies, *ESR* Erytrocyte Sedimentation Rate, *CRP* C-Reactive Protein, *CDAI* Clinical Disease Activity Index, *RAID* Rheumatoid Arthritis Impact of Disease, *ROAD* Recent-Onset Arthritis Disability index, *n.s.* non significant

### Clinical outcomes

**Fig. 2:**
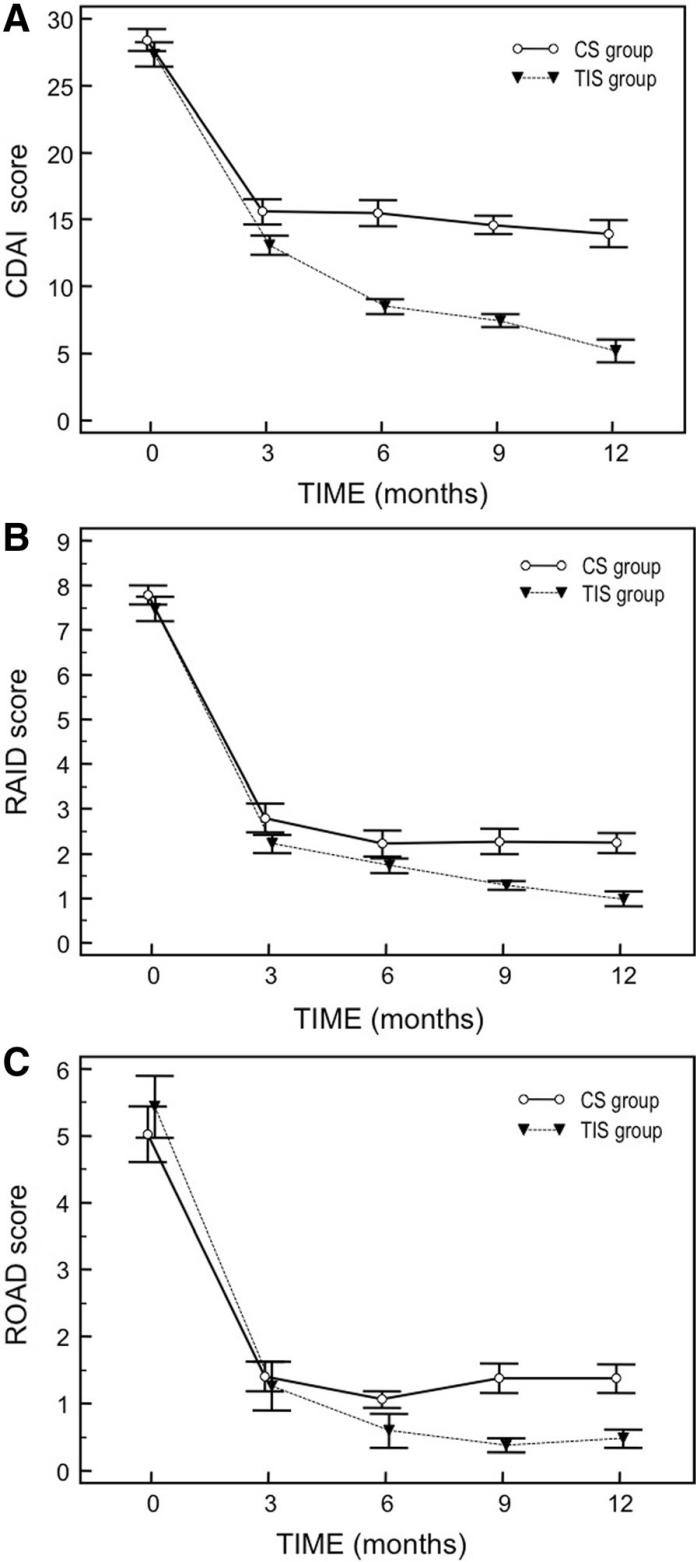
Median scores (standard error of the mean) of disease activity measured by CDAI (**a**) and RAID (**b**), and of functional disability by ROAD (**c**), at three-months intervals in TIS and CS groups

**Fig. 3:**
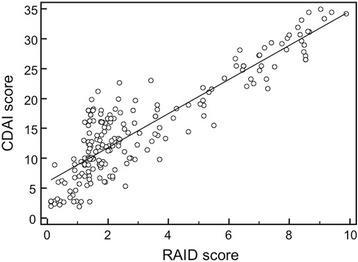
Scatter plot of CDAI (y-axis) and RAID (x-axis) values with a regression line

A higher percentage of patients in TIS achieved CDAI remission versus patients in CS (38.1 % vs 25 % at year 1, *P* <0.01). Time to achieve remission (CDAI <2.8) was significantly shorter in the TIS group than in the CS group, with a median of 20 weeks versus a median over 36 weeks (*P* <0.001). Figure 2 shows the mean scores of CDAI (± standard error of the mean – SEM) (Fig. 2a), RAID (Fig. 2b) and ROAD (Fig. 2c) at three-months intervals. All the clinical variables improved statistically significantly from baseline (*P* <0.001) in the TIS strategy group compared to the CS group. Already, after 12 weeks, the curves of the TIS group appeared to diverge (Fig. 2a-b-c). The results show also a good correlation between the scores of CDAI and the RAID (Spearman’s coefficient of rank correlation rho = 0.771, *p* < 0.0001) (Fig. 3), both measures of disease activity.

The comparison between the mean values of AUC and CDAI of both groups after 12 months resulted in favour of the TIS group in comparison with the CS group (145.22 ± 29.25 vs 180.23 ± 38.62, P = 0.003 respectively) (Table 2). The mean values of CDAI, obtained at the end of follow-up, were also much better in the TIS group than in the CS group (−73.76 ± 16.81 vs −57.33 ± 23.83, *P* = 0.011, respectively) (Table 2). The 1-year AUC of CDAI minor, moderate and major response cut points (50 %, 70 % and 85 % improvement) (27) were also studied. The comparison between the two groups of patients (Table 3) confirmed the better results in TIS vs those in the CS group. In particular, a major response (CDAI cut-off >85 %) was observed in 9 patients followed by TIS (42.8 %) compared with 5 patients (25 %) included in CS group.

**Table 2:** Summary measure of CDAI - Area under curve and percentage difference last-first

Area under curve					
Group	Mean	95 % CI	SD	Median	95 % CI
TIS	145.22	131.90 to 158.53	29.25	142.24	124.86 to 164.23
CS	180.23	162.15 to 198.31	38.62	192.24	159.02 to 209.15
Average rank of first group			15.66		
Average rank of second group			26.60		
Mann–Whitney U			98.00		
Large sample test statistic Z			2.92		
Two-tailed probability			*P* = 0.003		
Percentage difference last-first					
Group	Mean	95 % CI	SD	Median	95 % CI
TIS	−73.76	−81.41 to −66.10	16.81	−66.90	−90.09 to −62.62
CS	−57.33	−68.49 to −46.18	23.83	−57.43	−67.56 to −43.37
Average rank of first group			16.33		
Average rank of second group			25.90		
Mann–Whitney U			112.00		
Large sample test statistic Z			2.55		
Two-tailed probability			*P* = 0.011

**Table 3:** CDAI response cutpoints in the active treatment arms of TIS and CS patients (response status at 12-months)

CDAI response category	TIS (*n* = 21)	CS (*n* = 20)	*P*
No response, n° (%)	2 (9.6)	8 (40)	<0.001
CDAI 50, n (%)	9 (42.8)	7 (35)	<0.01
CDAI 70, n (%)	1 (4.8)	0	n.v.
CDAI 85, n (%)	9 (42.8)	5 (25)	<0.001

*n.v. = non valuable*

### Radiographic evaluation of joint damage

Of the 41 RA patients, 36.6 % were erosive at baseline, 58.5 % were erosive at the 12th month. The mean annual progression rate in the overall cohort, according to the TSS method, resulted in 5.5 ± 4.4 units. It can be seen that when progression of joint damage was compared across group patients, these in TIS showed a lower total radiographic progression in terms of delta-TSS than CS cohort (delta-TSS, TIS group vs CS group, 1.47 vs 2.70; *P* = 0.009). In particular, 5 patients (23.8 %), followed by TIS did not show disease progression (as defined by delta-TSS ≤0.5, since baseline) [22], whereas only 1 patient (10 %) was non-progressor in the CS group.

### Patients meeting all criteria for CDC at 1 year

**Fig. 4:**
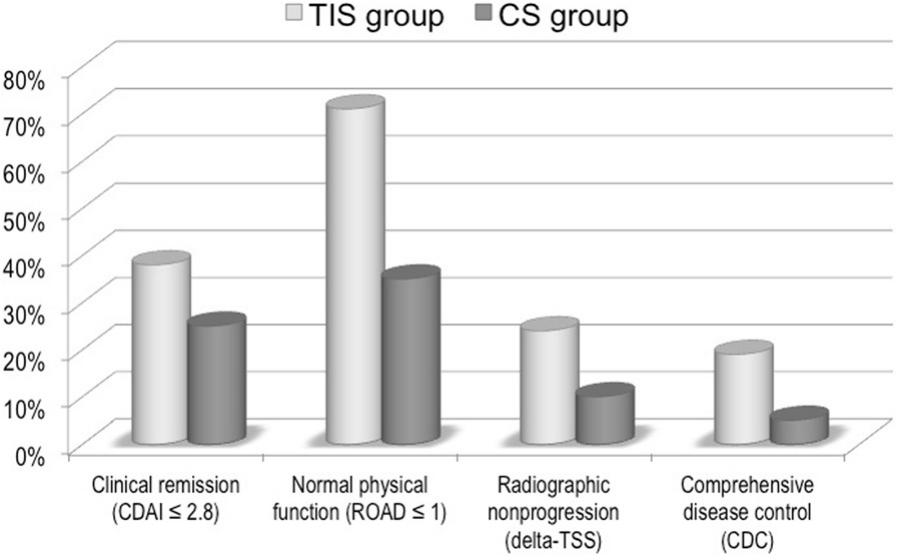
Proportions of patients who achieved the CDC, defined as clinical remission (CDAI ≤2.8), normal physical function (ROAD ≤1) and absence of radiographic progression (delta-TSS ≤0.5) at 1-year

Figure 4 shows the proportions of patients who achieved the CDC, defined as clinical remission (CDAI ≤2.8), normal physical function (ROAD ≤1) [36] and absence of radiographic progression (delta-TSS ≤0.5) [22]. The patients followed for one year in TIS showed a greater improvement (*P* <0.001) compared with the CS group in terms of clinical activity (38.1 % vs 25 %), functional impairment (71.4 % vs 35 %) and radiological joint damage progression (23.8 % vs 10 %), resulting in a greater rate of CDC (19.4 % vs 5 %).

### Patients’ satisfaction with the technological device

We asked the 21 participants who have completed the follow-up (TIS group) to indicate their satisfaction with the technological device at the end of the intervention. The results indicate high satisfaction among the vast majority of respondents, with an overall average of 4.28 on a scale of 5 (where 1 = not at all and 5 = enormously) (Table 4). Furthermore, in response to the question “If you could keep the automated telemonitoring system in your home, would you continue to use it in the future?”, 90.5 % said that they would.

**Table 4:** Patients’ satisfaction with the technological device

Questions	Average	S.D.
a) The automated telemonitoring system is easy to use	4.3	0.9
b) The automated telemonitoring system is easy to understand	4.4	1.1
c) The information in the telemonitoring system meets my personal needs	4.0	0.8
d) I find the information in the telemonitoring system useful for managing my own care	4.1	0.9
e) In general, I am satisfied with my use of the automated telemonitoring system	4.6	1.0

Scales of 1 to 5 where: 1 = not at all; 2 = somewhat; 3 = moderately; 4 = very; 5 = enormously

## Discussion

This is the first randomised controlled pilot study in an ERA population, evaluating whether a telemonitoring intensive approach, by a Web platform, was more effective than a conventional care strategy in reaching remission and CDC [22] after 1 year in daily clinical practice. We also reported the patients’ acceptance of this telemonitoring platform.

The key message of this paper is that a scheduled intensive strategy, based on a telemonitoring system, is a useful approach to achieve remission and CDC more often and faster than a conventional strategy. After 1 year, remission (CDAI <2.8) was reached in 38.1 % of patients in the TIS group as compared to 25 % of patients in the CS group. Time to achieve remission was significantly shorter in the TIS group than in the CS group, with a median of 20 weeks versus a median over 36 weeks (*P* <0.001). Concordantly, the TIS strategy resulted in a larger decrease of CDAI, more patients having low disease activity, and larger improvements in functional ability and radiological joint damage progression, resulting in a greater rate of CDC (19.4 % vs 5 %). Furthermore, we found that collection of subjective data using an electronic platform in this setting is a feasible method than can be adopted with high compliance rates across a range of RA patient.

In the present research, the mean percentage of remission achieved in ERA patients, according to CDAI criteria, was quite similar to that obtained in our previous study [40], and for the groups in the BeSt study [41], ESPOIR cohort [42], but lower than other tight control studies [31, 43–45]. Goekoop-Ruiterman et al. [46] and Schipper et al. [5] previously compared a tight control treatment with usual care and showed a better response after 1 year for tight control. Other data suggest that tight control may improve longterm functional disability [47], and reduce the need for joint surgery among patients with RA treated with biological agents [48].

Emery et al. [22] found that patients with RA who achieved CDC at week 26 by a tight control (a total of 19.4 %) had highly improved short-term and long-term health-related quality of life, pain, fatigue and work-related outcomes compared with patients who had not achieved CDC.

Although previous works experienced an intensive computed management [31, 49], the design of our study is unique and differs on several points from other studies: our intensification of treatment in the TIS group was based on the percentage change in disease activity with respect to each previous visit performed. This data was generated by the telemonitoring system. Specifically, if the RAID did not show an improvement of at least 30 % in the first two months of treatment (relative improvement) or 2 points (absolute improvement), then from the third month onwards at least a 50 % (relative improvement) or 3 points (absolute improvement), the system automatically suggested an appropriate required action to rheumatologist [34].

Computerized information can provide useful support in the process of shared decision-making. This concept emphasizes the role of the patient as an active partner of the physician in choosing the proper treatment [50]. Internet offers great potentials and new opportunities for e-communication, patient monitoring (telemonitoring), disease management and clinical research. A systematic review of experimental and quasi-experimental studies involving home telemonitoring of chronic patients revealed that home telemonitoring seems to be a promising patient management approach that produces accurate and reliable data, empowers patients, influences their attitudes and behaviors, and potentially improves their medical conditions [17]. A review of systematic reviews [51] concluded that telemedicine works has positive effects. These include therapeutic advantages, increased efficiencies in the health services and technical usability. “Telerheumatology” may also prove to be an effective tool for managing patients with RA [18]. Monser et al. found that rheumatological patients, have significantly increased their Internet activity [52]. Therefore, interactive Internet services including the patient perception and opinion may represent an effective and important option to contribute to the improvement of the disease [53].

Several reviewers suggested that telemedicine seemed to be cost-effective, but few drew firm conclusions. A particular limitation identified in terms of costs concerns the wider social and organisational costs of telemedicine in RA patients [54].

Some limitations of our study should be mentioned: first, the small sample size may have limited our ability to demonstrate suitability of telemonitoring as an alternative to face-to-face care. No sample-size calculation was made. Therefore, the results of this analysis have to be considered as hypotesis generating for future research. Second, the short duration of the study which may influence the results. However we believe that 1 year is a reasonable period to extrapolate preliminary data. Finally, only patients able to use Internet were involved. In a real situation, not all patients have confidence with Internet.

## Conclusions

The results of this study suggest the following conclusions: (a) a higher percentage of patients in TIS achieved CDAI remission versus patients in usual care (38.1 % vs 25 %); (b) time to achieve remission was significantly shorter in the TIS group than in the CS group; (c) the patients followed for one year in TIS showed a greater improvement compared with the CS group in terms of clinical activity, functional impairment, and radiological joint damage progression, resulting in a greater rate of CDC (19.4 % vs 5 %).

In addition to other tools, telemonitoring can contribute to an increased adherence to pharmacological and non-pharmacological recommendations among RA patients [55]. Furthermore, participating rheumatologists indicated that the telemonitoring system could be a helpful tool in their daily clinical practice. The findings of this work, due to the methodological approach of a pilot study, including a limited sample size, short duration of the assessment, as well as generalizability of the results, need to be confirmed with larger controlled studies.

### Availability of data and materials

Data coming from these two groups of patient will be not shared. They represent an inception cohort to generate further research on the telemonitoring strategy in the field of rheumatoid arthritis.

## Abbreviations

ABA: Abatacept
ACPAs: Anti-citrullinated proteins antibodies
ACR: American college of rheumatology
ADA: adalimumab
AUC: area under the curve
CDAI: clinical disease activity index
CDC: comprehensive disease control
CI: confidence interval
CS: conventional strategy
CRP: C-reactive protein
DMARD: disease modifying anti-rheumatic drugs
ERA: early rheumatoid arthritis
ESR: erythrocyte sedimentation rate
ETN: etanercept
EULAR: European league against rheumatism
ICC: intraclass correlation coefficient
LDA: low disease activity
MTX: methotrexate
NRS: numerical rating scale
NSAIDs: Non-steroidal anti-inflammatory drugs
PRO: patient reported outcome
RA: Rheumatoid arthritis
RAID: Rheumatoid arthritis impact of disease
RETE-MARCHE: REmote TElemonitoring for MAnaging Rheumatologic Condition and HEalthcare programmes
RF: Rheumatoid Factor
ROAD: recent-onset arthritis disability index
SD: standard deviation
SEM: standard error of the mean
SPSS: statistical package for social sciences
SSZ: sulphasalazine
T2T: treat-to-target
TIS: telemonitoring intensive strategy
TSS: total sharp score
VAS: visual analogue scale
